# Targeting Cholesterol Homeostasis to Fight Hearing Loss: A New Perspective

**DOI:** 10.3389/fnagi.2015.00003

**Published:** 2015-01-29

**Authors:** Brigitte Malgrange, Isabel Varela-Nieto, Philippe de Medina, Michael R. Paillasse

**Affiliations:** ^1^GIGA-Neurosciences, Developmental Neurobiology Unit, University of Liege, Liege, Belgium; ^2^Instituto de Investigaciones Biomédicas “Alberto Sols”, CSIC-UAM. IdiPAZ, CIBERER Instituto de Salud Carlos III. Arturo Duperier 4, Madrid, Spain; ^3^Affichem SA, Toulouse, France

**Keywords:** sensorineural hearing loss, cholesterol homeostasis, liver X receptor, excitotoxicity, oxysterol

## Abstract

Sensorineural hearing loss (SNHL) is a major pathology of the inner ear that affects nearly 600 million people worldwide. Despite intensive researches, this major health problem remains without satisfactory solutions. The pathophysiological mechanisms involved in SNHL include oxidative stress, excitotoxicity, inflammation, and ischemia, resulting in synaptic loss, axonal degeneration, and apoptosis of spiral ganglion neurons. The mechanisms associated with SNHL are shared with other neurodegenerative disorders. Cholesterol homeostasis is central to numerous pathologies including neurodegenerative diseases and cholesterol regulates major processes involved in neurons survival and function. The role of cholesterol homeostasis in the physiopathology of inner ear is largely unexplored. In this review, we discuss the findings concerning cholesterol homeostasis in neurodegenerative diseases and whether it should be translated into potential therapeutic strategies for the treatment of SNHL.

## Introduction

Hearing loss constitutes a major health problem affecting 16% of the adult population worldwide (Pleis and Lethbridge-Cejku, 2006). Aging is the main risk factor associated with hearing impairment. Age-related sensorineural hearing loss (SNHL) is the third most common disability of the elderly affecting about half of the population over 75 years old (Gates and Mills, 2005). SNHL prevalence dramatically increases and is expected to keep rising based on the rapidly increasing number of elderly people. SNHL is a pathology of the cochlea that is generally regarded as mechanical or chemical damage-induced hair cell death triggering spiral ganglion neuron (SGN) death and subsequent dysfunction of auditory nerve (Takeno et al., 1998). Recent researches in SNHL field have lead to a more complex vision of the relationship between inner ear damage and SNHL. Indeed, SGN loss without hair cell damage or death was observed (Ryals et al., 1999; White et al., 2000; Linthicum and Fayad, 2009). Because many cell types within the cochlea, including hair cells, SGN, and strial cells, decrease in number with age (Ohlemiller and Gagnon, 2004), the majority of age-related SNHL could be classified according to the type of cell degenerated: sensory (hair cell loss), neural (SGN loss), metabolic (strial dysfunction), and cochlear conductive (changes in the stiffness of the basilar membrane) (Schuknecht and Gacek, 1993). Consistent with this, auditory neuropathy and auditory synaptopathy were reported as a cause of SNHL. Auditory synaptopathy results from defects of the ribbon synapses between inner hair cells and SGN (Moser et al., 2013) leading to auditory neuropathy that is characterized by auditory nerve degeneration (Worthington and Peters, 1980; Starr et al., 1996). Auditory neuropathy is responsible for about 8% of SNHL cases and is notably associated with absent or abnormal ABR and poor speech understanding, particularly in noisy surroundings (Starr et al., 1996; Kraus et al., 2000; Madden et al., 2002).

Currently, no effective medication is available to prevent or treat SNHL. Cochlear implants bypass damaged hair cells by providing direct electrical stimulation of SGNs. This approach ameliorates speech production and perception in patients with a severe-profound SNHL (Harris et al., 1995; Bond et al., 2009). However, the beneficial effects of cochlear implants are strongly limited by both SGN degeneration and loss (Roehm and Hansen, 2005; Shibata et al., 2011). The neurotrophic and neuroprotective properties of neurotrophins were promising. However, first clinical trials led to variable results, showed bad distribution profiles and deleterious secondary effects such as abnormal proliferation of Schwann cells (Winkler et al., 1997), unwanted cell migration (Williams, 1991), or weight loss (Eriksdotter Jonhagen et al., 1998). Other trophic factors have shown effectiveness in modulating inner ear protection and repair, such as of insulin-like growth factor 1 (IGF-1). IGF-1 is effective in the protection from electrode trauma insertion in the guinea pig and in the recovery from sudden hearing loss in humans (Kikkawa et al., 2014; Nakagawa et al., 2014). This is promising, since, in men and mice, IGF-1 deficiency causes SNHL (Varela-Nieto et al., 2013) but more trials are needed. During the past few decades, other key mechanisms contributing to SNHL etiology were characterized. Indeed, noise-induced and age-related SNHL etiology was associated with ischemia, inflammation, excitotoxicity (excessive glutamate release), axonal degeneration, oxidative stress, and mitochondrial dysfunction (Menardo et al., 2012). Circulatory disturbance is considered as a plausible cause of idiopathic sudden SNHL (Kim, 1999; Merchant et al., 2008). Ischemia by itself causes excitotoxicity, failure of energy supply, and excess production of free radicals highlighting the interconnection between these deleterious processes. Excitotoxicity is also considered as a major mediator of inner ear damage leading to deleterious effect on SGN function. New therapeutic approaches that target several of these deleterious processes should be effective for SNHL prevention and treatment.

Besides SNHL, these deleterious processes are also causative or characteristic factors of neurodegenerative diseases. Interestingly, cholesterol homeostasis and metabolism are central to numerous pathologies including neurodegenerative diseases (Liu et al., 2010; Vance, 2012) and regulate the above-mentioned processes involved in neuron survival and functionality (Laskowitz et al., 1997; Kang and Rivest, 2012). Consequently, interfering with cholesterol homeostasis should afford innovative therapeutic strategies to improve the care of SNHL. In this review, we discuss the underestimated potential of cholesterol homeostasis and metabolites as a new opportunity to better understand inner ear pathologies and afford innovative therapeutic strategies.

## Cholesterol Homeostasis in Brain

**Figure 1:**
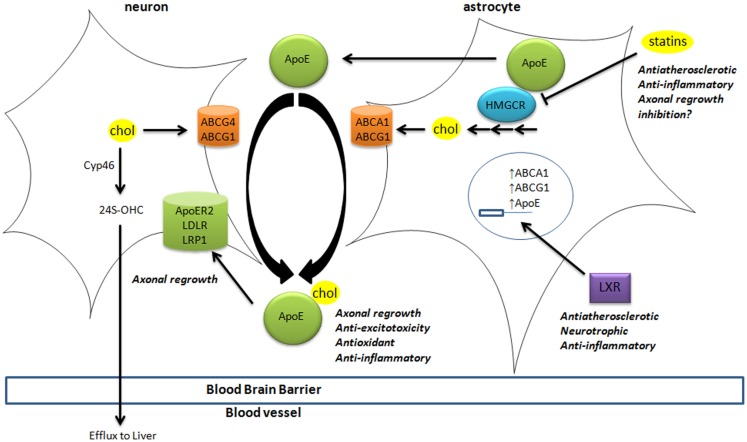
**Cholesterol homeostasis in brain**. Cholesterol synthesis takes place in astrocytes, through activation of the rate-limiting enzyme HMGCR. Cholesterol is then loaded on ApoE particles by ABCA1 and ABCG1 transporters. LXR activation triggers the expression of ApoE, ABCA1, and ABCG1 at the transcriptional level. These lipoproteins are internalized by neurons via LDL-family receptors (LDLR, LRP1, and ApoER2). In neurons, cholesterol is metabolized into 24(S)-OHC by Cyp46 to be excreted through the blood–brain barrier to the liver. The impact of key players in cholesterol homeostasis (HMGCR, LXR, ApoE-lipoproteins, and LRP1) in processes associated with neurodegeneration is disclosed (italic). 24(S)-OHC, 24(S)-hydroxycholesterol; ABC, ATP-binding cassette; ApoER2, ApoE receptor 2; Cyp46, cytochrome P450 46A1 or cholesterol-24-hydroxylase; HMGCR, HMGCoA reductase; LDL, low density lipoprotein; LDLR, low density lipoprotein receptor; LRP1, LDL-related protein 1; LXR, liver X receptor.

Brain cholesterol is essential to ensure cell membrane structure, neurotransmitter release, signal transduction, and synaptogenesis (Pfrieger and Ungerer, 2011; Leoni and Caccia, 2013). Since the blood–brain barrier (BBB) prevents the uptake of lipoprotein from the circulation, all brain cholesterol is synthesized from acetyl-CoA through the rate-limiting enzyme HMGCoA reductase (HMGCR), tightly regulated by sterol-regulator element binding protein (Figure 1). In adult brain, neurons mostly rely on cholesterol from astrocytes, secreted by adenosine triphosphate-binding cassette (ABC) members A1 and G1, and bound to apolipoprotein E (ApoE) particles. Neurons then uptake these lipoproteins via receptors of the low density lipoprotein receptor family (i.e., LDL receptor, LDL receptor-related protein 1, and ApoE receptor 2). Cholesterol is notably required to form synapses (Goritz et al., 2002) in neuronal cells. Excess cholesterol is converted by Cyp46 into 24(S)-hydroxycholesterol [24(S)-OHC], then secreted directly or via ABCG4 to ApoE particles. Contrary to cholesterol, some oxysterols are able to cross the BBB, since 24(S)-OHC is excreted to circulation whereas 27-hydroxycholesterol (27-OHC) reaches the brain (Figure 1).

These oxysterols fluxes are important since most of those are endogenous ligands of liver X receptors (LXRs) (Janowski et al., 1996; Fu et al., 2001). LXRα and LXRβ are nuclear transcription factors that are master regulators of cholesterol homeostasis (Hong and Tontonoz, 2014), regulating the expression of the above-mentioned cholesterol transporters (Figure 1). For instance, the expression of ABCA1 and ABCG1 was reduced in astrocytes from LXR-invalidated mice, and LXR was shown to be essential for neurogenesis (Fan et al., 2008). Some oxysterols that are LXR ligands were detected in brain and display neurotrophic activity *in vitro* and *in vivo* (Schmidt et al., 1999; Sacchetti et al., 2009; Theofilopoulos et al., 2013).

Cholesterol homeostasis in the inner ear is largely unexplored. However, it is highly probable that similar mechanisms may rule cholesterol homeostasis in brain and cochlea. Indeed, neither brain nor cochlea can use cholesterol from the circulation and expression of cholesterogenic enzymes, cholesterol transporters, and LXR was reported in both.

## Cholesterol Homeostasis and Neurodegenerative Disease

Deregulation of cholesterol balance is an increasingly recognized characteristic of chronic neurodegenerative diseases such as Parkinson’s, Alzheimer’s, and Huntington’s diseases (Vance, 2012). Acute neuronal injury in stroke, brain trauma, or epileptic seizures also impact cholesterol homeostasis in the brain (Mahley, 1988; Adibhatla and Hatcher, 2008). Changes in brain cholesterol homeostasis were described during glutamate-mediated excitotoxicity, which is involved in the deleterious effect of numerous neurological stresses such as stroke, traumatic brain injury, and noise exposure. Nevertheless, roles of cholesterol and its metabolites are not clear (Ong et al., 2010; Sodero et al., 2012). Short-term glutamate mediated excitotoxicity induces a cholesterol loss from the synaptic membranes through the stimulation of 24(S)-OHC production potentially leading to excitotoxicity attenuation since cholesterol and oxysterols (notably 7-ketocholesterol and cholesterol epoxides, 7KC and 5,6-ECs, respectively) are promoters of exocytosis. Other study showed that longer exposure to a potent glutamate analog lead to increased level of cholesterol and oxysterols (notably 7KC and 5,6-ECs) in neurons of the damaged hippocampus that potentially propagate excitotoxicity and directly induce cytotoxicity. Consistently, inhibition of cholesterol synthesis by statins or depletion by methyl-β-cyclodextrin prevents excitotoxicity-induced neuronal death (Ponce et al., 2008).

On the other hand, cholesterol derived from astrocytes lipoprotein seems beneficial in neurons. Indeed, in glial cells, dramatic increase of ApoE produced was described after nerve injury in both central and peripheral nervous systems (Ignatius et al., 1986; Boyles et al., 1989), allowing axonal regrowth, and repair of injured neurons as shown in retinal ganglion neurons (Hayashi et al., 2004). An upregulation of ABCA1 was also observed *in vivo* during reinnervation of damaged hippocampus (Jasmin et al., 2014). In neurons, LDL receptor family supports ApoE beneficial action (Hayashi et al., 2004). For instance, LRP1 activation promotes axonal regeneration (Yoon et al., 2013) and induces neurotrophin receptor signaling (Shi et al., 2009). Altogether, these studies showed that ApoE-lipoproteins exert antioxidant, anti-inflammatory, and anti-excitotoxic activities and stimulate axonal regrowth by providing cholesterol to distal axons.

Numerous studies sustain the beneficial impact of LXR in neurodegeneration and as target for neuroprotective/regenerative treatments. LXR receptors disruption in mice is associated with severe neurodegeneration (Wang et al., 2002). The brain of LXR-invalidated mice displayed enlarged brain blood vessels, lipid deposits, proliferation of astrocytes, and loss of neurons. The impairment of cholesterol delivery from astrocytes to neurons should be a major cause of neurodegeneration observed in the LXR-invalidated mice. Consistently, LXR activation using synthetic ligands improves recovery in a rat model of acute brain ischemia (Namjoshi et al., 2013). In addition to homeostasis, role of LXR in inflammation is also major in diverse pathologies including neurodegenerative diseases (Steffensen et al., 2013). LXR activation prevents the transcription of inflammatory genes through the inhibition of NFκB pathway. In addition, synthetic LXR agonists reduce neuroinflammation in mice models of neurodegeneration and exert neuroprotective property *in vivo* (Sironi et al., 2008). Interestingly, some endogenous oxysterols do so *in vitro* and *in vivo* (Schmidt et al., 1999; Sacchetti et al., 2009; Theofilopoulos et al., 2013).

**Figure 2:**
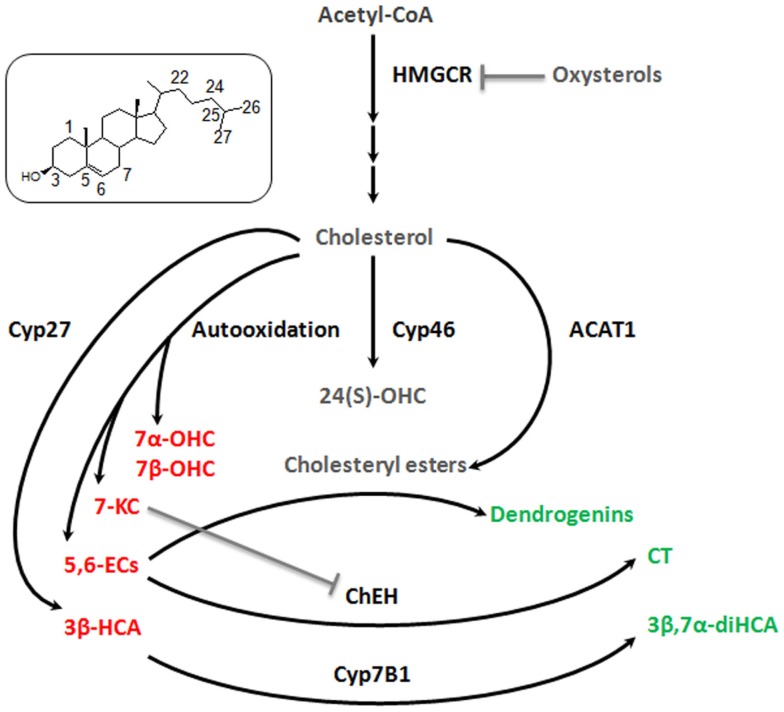
**Good and bad cholesterol metabolites in brain**. As it was described decades ago for cardiovascular diseases and more recently for cancer, it appears that there is a balance between good and bad cholesterol in neurodegeneration process. Cholesterol is oxidized by different ways (enzymatic or auto-oxidation) to give rise to a large number of oxysterols. Some appear to be bad, as they stimulate exocytosis, excitotoxicity, apoptosis (7α-OHC, 7β-OHC, 5,6α-EC, 5,6β-EC, and 7KC, 3β-HCA – in red), some appear to be good as they exert neuroprotective, neurotrophic, or anti-inflammatory activities (CT, 3β,7α-diHCA, dendrogenins – in green) and finally some whose role is not clear, such as 24(S)-OHC and CE for which additional researches will be necessary to fully understand their involvement in neuroprotective or neurodegenerative processes (in gray). 7α-OHC, 7alpha-hydroxycholesterol; 7β-OHC, 7beta-hydroxycholesterol; 7KC, 7-ketocholesterol; 5,6α-EC, 5,6alpha-epoxycholesterol; 5,6β-EC, 5,6beta-epoxycholesterol; CT, cholestane-3β,5α,6β-triol; 3β,7α-diHCA, 3β,7α- dihydroxycholest-5-en-26-oic acid; 3β-HCA, 3β-hydroxycholest-5-en-26-oic acid; 24(S)-OHC, 24-hydroxycholesterol. Cyp27, cytochrome P450 27A1; Cyp46, cytochrome P450 46A1; Cyp7B1, cytochrome P450 7B1; ACAT-1, Acyl-CoA cholesterol acyltransferase; ChEH, cholesterol epoxide hydrolase; HMGCR, HMGCoA reductase.

The studies related to brain cholesterol metabolites have essentially focused on 24(S)-OHC. It presents a Janus face, namely, the induction of cell death at high concentration (above 10 μM) and, at lower doses, an adaptive protective response against cytotoxic oxysterols. The former is due to increased exocytosis that may aggravate excitotoxic injury (Ma et al., 2010). The latter results from a stimulation of an LXR-dependant increase of ABCG1 that should promote the efflux of cytotoxic oxysterols formed during oxidative stress (Noguchi et al., 2014). Some observations describe other oxysterol players in the brain. 7alpha-hydroxycholesterol (7α-OHC), 7beta-hydroxycholesterol (7β-OHC), 5,6alpha-epoxycholesterol (5,6α-EC), 5,6beta-epoxycholesterol (5,6β-EC), and 7KC are self-oxidation products of cholesterol that were detected in rat hippocampus (Figure 2). The level of these oxysterols was strongly increased after excitotoxicity (Ong et al., 2010). They increase exocytosis, intracellular calcium concentrations, and cytotoxicity (in particular 7KC), and could so propagate excitotoxicity. Cholestane-3β,5α,6β-Triol (CT) was found in rat brain (Hu et al., 2014). This oxysterol is produced by the hydrolysis of 5,6α-EC and 5,6β-EC catalyzed by the cholesterol epoxide hydrolase (ChEH) enzymatic activity (De Medina et al., 2010). CT exhibits neuroprotective activity both *in vitro* and *in vivo* (Figure 2). Indeed, this oxysterol protects against glutamate-induced cytotoxicity and decreased neuronal injury in different animal models. These beneficial effects may stem from the ability of CT to bind and negatively modulate NMDA receptors. Moreover, CT level was increased with ischemic preconditioning and the subsequent neuroprotective effect were abolished by an inhibitor of ChEH. It is noteworthy that 5,6-ECs and 7KC that display neurotoxic effect are, respectively, substrates and inhibitor of ChEH suggesting a potential pathophysiological inter-relation between these oxysterols that have opposite effect on neurons.

Cholestenoic acids, intermediates in the metabolism of cholesterol to bile acids, are present in neural tissues. Among cholestenoic acids, 3β,7α-dihydroxycholest-5-en-26-oic acid and 3β-hydroxycholest-5-en-26-oic acid regulate motor neuron function. 3β,7α-dihydroxycholest-5-en-26-oic acid promoted motor neuron survival in an LXR-dependant manner whereas 3β-hydroxycholest-5-en-26-oic acid triggers motor neuron loss (Theofilopoulos et al., 2014). These observations suggest a metabolic balance at the level of cholestenoic acids that may influence neurons fate (Figure 2). Cholesteryl esters (CEs) were detected in the brain (Martin and Bazan, 1992; Mulas et al., 2005). CEs are produced by the esterification of cholesterol with fatty acids catalyzed by Acyl-CoA: cholesterol acyltransferase (ACAT). CEs and ACAT-1 levels are increased in aging brain and in brain lesions. Moreover, increased expression of ACAT-1 and CEs level were reported in the hippocampus after excitotoxicity injury (Kim et al., 2011). Since excitotoxicity is associated with the production of cytotoxic oxysterols, esterification should sequester cholesterol to avoid this deleterious process. Conversely, cholesterol storage could also be deleterious by limiting the pool of cholesterol necessary to axonal regrowth, lipid raft functionality, and ApoE-lipoprotein delivery to neurons (Cutler et al., 2002). Whether CEs accumulation constitutes a neuroprotective response or participates in neuronal damage remains to be elucidated.

We previously reported that synthetic steroidal alkaloids resulting from the condensation of biogenic amines and 5,6α-epoxysterols display remarkable neurotrophic and neuroprotective activity *in vitro* (De Medina et al., 2009). Two of the most effective steroidal alkaloids identified to date are 5α-hydroxy-6β[2-(1H-imidazol-4-yl)ethylamino]cholestan-3β-ol, or dendrogenin A and 5α-hydroxy-6β-[3-(4-aminobutylamino)propylamino] cholest-7-en-3β-ol, or dendrogenin B. Dendrogenin B also promotes motor neuron survival (De Medina et al., 2009). In addition, these compounds induce proliferation and differentiation of neural stem cells (Khalifa et al., 2014). Dendrogenin A was recently characterized as a metabolite of 5,6α-EC in mammal tissues, including brain (De Medina et al., 2013). Thus, dendrogenins could be involved in the maintenance of nerve functional state including in inner ear.

As illustrated in Figure 2, cholesterol conversion in the brain is a double edged sword that can generate good or bad metabolites. A similar situation was reported for cancer (Silvente-Poirot and Poirot, 2014). This cholesterol balance should be involved in the normal and pathological physiology of the inner ear. To our knowledge, cholesterol metabolism has never been precisely studied in the inner ear.

## Cholesterol Homeostasis and SNHL

Even if studies related to cholesterol homeostasis in inner ear are scarce, some reports support a relationship between cholesterol homeostasis deregulation and SNHL. Indeed, the genetic syndromes Niemann–Pick type C and Smith–Lemli–Opitz that affect, respectively, cholesterol intracellular transport and synthesis display devastating neurological phenotypes including SNHL (Di Berardino et al., 2007; King et al., 2014). Some epidemiology studies revealed that hypercholesterolemia predisposes to SNHL (Suzuki et al., 2000; Weng et al., 2013). Indeed, atherosclerosis, high plasma total cholesterol, and low HDL levels are positively correlated with SNHL. Medication used for prevention and treatment of atherosclerosis such as Simvastatin were described as otoprotective in mice (Cai et al., 2009). Consistently, ApoE knockout mice developed marked hyperlipedimia, atherosclerosis, and hearing impairment (Guo et al., 2005). The most plausible explanation is that hypercholesterolemia triggers the stenosis of spiral modiolar artery leading to cochlear ischemia and subsequent SNHL. Consequently, therapies that limit high plasma cholesterol level could be useful to prevent SNHL caused by cochlear ischemia.

## Therapeutic Perspectives

Cholesterol homeostasis and metabolism play an important role in neurodegenerative disease and interfere with major causative processes, which are also strongly associated with SNHL, suggesting that targeting cholesterol homeostasis should provide innovative strategies to prevent and attenuate SNHL (Figure 1). On this basis, we proposed some hypothesis to be explored for SNHL treatment.

Statins (HMGCR inhibitors) have been proposed as treatment for neurogenerative diseases including SNHL notably through anti-atherosclerotic effect on cochlear artery and anti-inflammatory activity. Cholesterol-lowering agents should be useful to prevent ischemia and subsequent SNHL. However, this approach should be limited since damaged SGN need cholesterol from astrocyte-derived ApoE-lipoproteins for axonal regrowth. It might be preferable to use cholesterol-lowering agent not crossing the BBB at least in already damaged inner ear. An interesting approach might be a treatment with LXR agonists. These compounds also prevent atherosclerosis via the stimulation of cholesterol efflux rather than direct effect on cholesterogenesis. In the inner ear, LXR agonists might also promote axonal regrowth of SGN by inducing ApoE-lipoprotein formation in astrocytes. In addition, LXR agonists exhibit direct neurotrophic effect *in vitro* and anti-inflammatory activity. However, LXR ligands biological properties are closely related to their structure and to the cell types. For example, the 3β,7α-dihydroxycholest-5-en-26-oic acid and 3β-hydroxycholest-5-en-26-oic acid that both target LXR are, respectively, neuroprotective and neurotoxic (Theofilopoulos et al., 2014). This event is associated with differential recruitment of coactivators/corepressors and subsequent regulation of gene-expression patterns, which strongly depend on the structure of LXR/ligand complex (Huang et al., 2010). The discovery of the bona fide LXR ligand for SNHL treatment remains a difficult challenge. ApoE possesses antioxidant, anti-inflammatory, anti-excitotoxic, and neurotrophic properties and has been proved to be effective in treating brain injury in multiple mouse models. Consequently, it is plausible that ApoE or ApoE mimetics have beneficial effect for the prevention or the treatment of SNHL. Since LRP1 agonist exhibits axonal regrowth properties, this approach should also be considered.

This review highlights the good and the bad side of cholesterol metabolites in neurodegenerative diseases (Figure 2). First of all, the determination of the endogenous level of these cholesterol metabolites in healthy and damaged inner ear should be informative. The effect of the good cholesterol metabolites (i.e., CT, 3β,7α-dihydroxycholest-5-en-26-oic acid, dendrogenins) should be investigated in animal models of SNHL (aminoglycosides or noise exposure, presbycusis). Another approach that deserves to be studied is the blockage of bad cholesterol metabolites (i.e., 5,6-ECs, 7KC, 3β-hydroxycholest-5-en-26-oic acid). Since these oxysterols are mainly produced by auto-oxidation, the use of antioxidants seems sensible. Antioxidants have been extensively investigated and are suitable preventive agents for SNHL. At the level of cholesterol metabolism, it is probable that antioxidants block the production of both good and bad cholesterol metabolites potentially limiting their efficacy. It is noteworthy that 5,6-ECs and 3β-hydroxycholest-5-en-26-oic acid are converted, respectively, by ChEH and Cyp7B1 to produce CT and 3β,7α-dihydroxycholest-5-en-26-oic acid previously described as neuroprotective. Pharmacological interventions that stimulate ChEH and Cyp7B1 should be useful. However, concerning ChEH, the situation is more complex since CT will be formed at the expense of dendrogenins biogenesis that also arises from enzymatic transformation of 5,6α-EC (Figure 2). Gevokizumab, an antibody targeting pro-inflammatory cytokine IL1β is under clinical evaluation, for treatment of autoimmune inner ear disease. Development of antibodies against bad cholesterol metabolites is also a potential alternative for SNHL. Despite the impact of 24(S)-OHC and cholesterol esterification in neurodegenerative diseases remain unclear (Figure 2), their effect in the inner ear also deserve to be studied.

## Concluding Remarks

This review proposes that the study of cholesterol homeostasis in the inner ear might afford new unexplored possibilities for the prevention and treatment of SNHL. Important tasks have to be done to achieve this aim. First: to characterize cholesterol homeostasis and metabolome in normal, aged, and damaged inner ear. Second: to determinate the impact of intervention of cholesterol homeostasis in SNHL. Third: to investigate whether cholesterol metabolites prevent, delay, or aggravate SNHL.

## Conflict of Interest Statement

Philippe de Medina and Michael R. Paillasse are employees of Affichem Company. They are inventors of two patents in relation (among others) with neuroprotection/neuronal differentiation induced by aminooxysterols.
